# Impact of program/erase operation on the performances of oxide-based resistive switching memory

**DOI:** 10.1186/s11671-014-0721-2

**Published:** 2015-02-05

**Authors:** Guoming Wang, Shibing Long, Zhaoan Yu, Meiyun Zhang, Yang Li, Dinglin Xu, Hangbing Lv, Qi Liu, Xiaobing Yan, Ming Wang, Xiaoxin Xu, Hongtao Liu, Baohe Yang, Ming Liu

**Affiliations:** Lab of Nanofabrication and Novel Device Integration, Institute of Microelectronics, Chinese Academy of Sciences, Beijing, 100029 China; Tianjin Key Laboratory of Film Electronic and Communication Devices, Tianjin University of Technology, Tianjin, 300384 China

**Keywords:** Resistive random access memory (RRAM), Current sweep, Pulse operation, Uniformity, Endurance, Weibull distribution

## Abstract

Further performance improvement is necessary for resistive random access memory (RRAM) to realize its commercialization. In this work, a novel pulse operation method is proposed to improve the performance of RRAM based on Ti/HfO_2_/Pt structure. In the DC voltage sweep of the RRAM device, the SET transition is abrupt under positive bias. If current sweep with positive bias is utilized in SET process, the SET switching will become gradual, so SET is current controlled. In the negative voltage sweep for RESET process, the change of current with applied voltage is gradual, so RESET is voltage controlled. Current sweep SET and voltage sweep RESET shows better controllability on the parameter variation. Considering the SET/RESET characteristics in DC sweep, in the corresponding pulse operation, the width and height of the pulse series can be adjusted to control the SET and RESET process, respectively. Our new method is different from the traditional pulse operation in which both the width and height of program/erase pulse are simply kept constant which would lead to unnecessary damage to the device. In our new method, in each program or erase operation, a series of pulses with the width/height gradually increased are made use of to fully finish the SET/RESET switching but no excessive stress is generated at the same time, so width/height-controlled accurate SET/RESET can be achieved. Through the operation, the uniformity and endurance of the RRAM device has been significantly improved.

## Background

Thanks to the increasing demand from portable electronic products like smartphones, cameras, and laptops, the demand for solid-state memories has been increasing rapidly in recent years. However, in the further scaling down, the traditional flash memory is facing more and more problems due to its physical limitations. Although innovations in cell structure and device materials may help extend flash memory for another couple of technology nodes, alternative candidates must be explored for future non-volatile memory (NVM) applications. Among various candidates, resistive random access memory (RRAM) is the most promising one for future high-density NVM application, owning to its characteristics of simple cell structure, fast program/erase (P/E) speed, excellent scalability, low operation power consumption, and good compatibility with the standard complementary metal-oxide-semiconductor (CMOS) process [1-5]. However, in order to meet the practical application requirements, the performances of RRAM demonstrated to date still need improvements in the following areas: (1) effective control of high and low resistance state; (2) minimization of the variations of resistive switching parameters. Some approaches have been proposed to improve the operation test method of RRAM. Nevertheless, few works systematically studied the detailed influence of DC and pulse program/erase operations on the performances of oxide-based RRAM. In this work, aiming at addressing the above challenges, we try to elucidate the impact of P/E operation on the performances through comprehensive device characterizations and to explore possible solutions through innovations in test and operation methods.

For the necessity of plenty of DC and pulse measurement, stable valence change mechanism (VCM) devices with Ti/HfO_2_/Pt structure [5-12] is made use of in this work. In the DC positive voltage sweep the SET transition is abrupt, but it becomes gradual under current sweep. The RESET process is gradual in negative voltage sweep. So SET and RESET are current and voltage controlled, respectively. Combining positive current sweep SET and corresponding negative voltage sweep RESET operation, stable and uniform distributions of on-state and off-state resistance can be obtained. Gaining inspiration from the DC SET/RESET characteristics, we proposed a novel pulse operation scheme, i.e. the width and height of the pulse series are adjusted to control the SET and RESET process, respectively. Our new method is different from the traditional pulse operation with single constant program/erase pulse. Thus, accurate SET/RESET controlled by pulse width/height can be achieved through our new method. As a result of the new method, the uniformity and endurance of the RRAM device has been significantly improved.

## Methods

Resistive switching memory devices with Ti/HfO_2_/Pt structure were fabricated as follows. First, after the standard chemical cleaning of the silicon substrate, a SiO_2_ film with a thickness of 100 nm was thermally grown through dry oxidation method. Then, Ti/Pt bilayer with thickness of 30/70 nm was sequentially deposited by e-beam evaporation to act as the bottom electrode (BE). Next, a high-quality 8-nm-thickness HfO_2_ resistive switching layer was grown by atom layer deposition (ALD) technology, which has the advantage of well controlling on the deposition parameters and excellent deposition uniformity. Finally, the 10/70-nm-thickness Ti/Pt bilayer or 70-nm-thickness Cu film was prepared by e-beam evaporation and then patterned by lift-off process to form the top electrode (TE). The area of TE is defined as 100 × 100 μm^2^. The DC electrical characteristics of the devices were measured by Keithley 4200-SCS semiconductor characterization system, where the Pt BE was grounded while the bias voltage was applied on the Ti/Pt or Cu TE. In the traditional pulse measurement, a single pulse was usually employed to fulfill the SET/RESET operation, and a small read pulse or a visual tool was used to verify if the SET/RESET is completed and to measure the switching time. In the height/width-adjusting pulse operation measurement, Keithley 4205-PG2 pulse generator was used to generate program/erase pulse series by an automatic procedure, and the device states were read by Keithley 4200-SCS. A matrix Keithley 707A is used to carry out the switching between the pulse program/erase operation and DC read operation.

## Results and discussion

**Figure 1:**
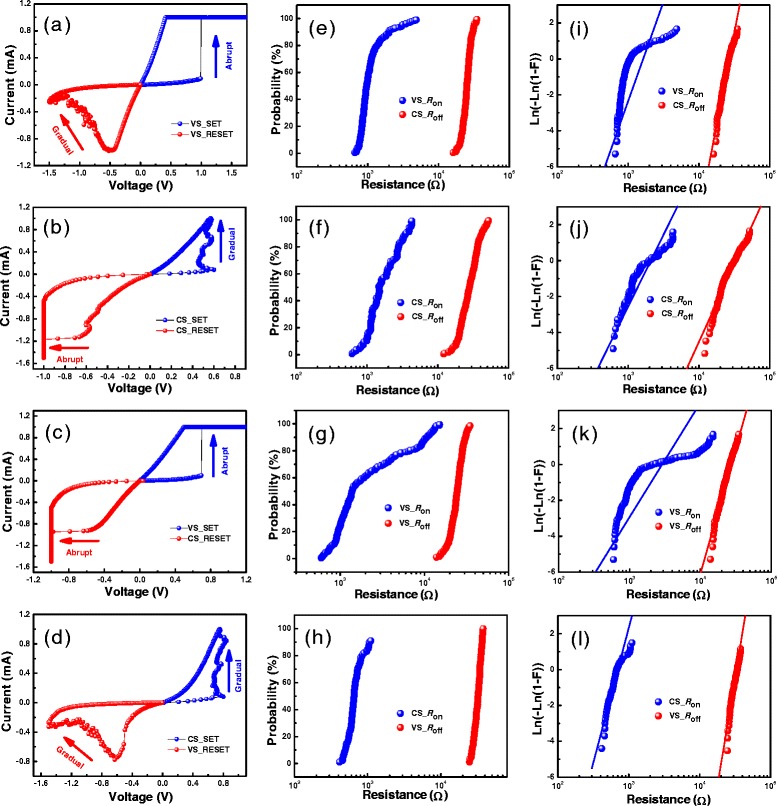
**Typical** ***I-V*** **curves and statistical distributions of Ti/HfO**_**2**_ **/Pt RRAM device under different operation. (a)** Positive voltage sweep SET and negative voltage sweep RESET processes. **(b)** Positive current sweep SET and negative current sweep RESET processes. **(c)** Positive voltage sweep SET and negative current sweep RESET processes. **(d)** Positive current sweep SET and negative voltage sweep RESET processes. **(e-h)** The cumulative distributions of *R*_on_ and *R*_off_ in 200 continuous cycles tested by the operation modes in **(a-d)**, respectively. **(i-l)** The Weibull plots of the distributions of *R*_on_ and *R*_off_ in correspondence with **(e-h)**, respectively. The straight lines are the lines fitting to standard Weibull distribution.

Figure 1a-d shows the *I-V* curves under four types of DC sweep measurement of the Ti/HfO_2_/Pt VCM device. The device works in bipolar switching mode, i.e. SET occurs in positive polarity while RESET is in negative bias. In the DC voltage sweep (VS) of the RRAM device (Figure 1a), the SET transition is abrupt under positive bias, while the change of current with applied voltage in RESET process is gradual in the negative voltage sweep, so the RESET operation is a voltage-controlled procedure. In the DC current sweep (CS) as shown in Figure 1b, it is in the opposite that the SET switching is a gradual so it is a current-controlled procedure. Since the abrupt SET process happens acrimoniously, it will produce large overshoot current, leading to great damage to RRAM device. Nevertheless, the gradual process will reduce the overshoot current and the controllability on the resistance value is easy to be achieved. Consequently, by combining the positive current sweep SET and the corresponding negative voltage sweep RESET operation, i.e. by integrating current-controlled SET and voltage-controlled RESET, as shown in Figure 1d, we can obtain an effective way to make the SET and RESET process to gently and gradually evolve. From Figure 1c, both abrupt SET and RESET processes are obtained by positive voltage sweep SET and corresponding negative current sweep RESET operation.

The statistical distributions of on-state and off-state resistance with different DC SET/RESET operation were studied. Figure 1e-h shows the cumulative distributions of *R*_on_ and *R*_off_ in 200 continuous cycles measured with the operation modes in Figure 1a-d, respectively. Comparing the results shown in Figure 1e-h, the distribution of *R*_on_ acquired by current-controlled SET is more uniform than that got by voltage sweep SET. At the same time, the uniformity of *R*_on_ and *R*_off_ measured by CS-SET and VS-RESET is also improved compared with that by CS-SET and CS-RESET. These results can also be seen from Table 1. The coefficient of variation (*σ*/*μ*) of *R*_on_ decreases from 52.2% in Figure 1f to 23.4% in Figure 1h. Moreover, *σ*/*μ* of *R*_off_ has also improved from 30.6% to 11.1%. The Weibull distribution is widely used in reliability forecast and evaluation [13-18]. The Weibull distribution is described by *F* = 1 − exp[−(*x*/*x*_63%_)^*β*^], where the parameter *x*_63%_ is the scale factor which is the value of the statistical variable at *F* ≈ 63%, *β* is the shape factor or Weibull slope which represents the statistical dispersion. Higher value of *β* means the tighter distribution of parameter, corresponding to the lower value of *σ*/*μ* in the normal distribution [19-21]. Figure 1i-l exhibits the Weibull plots of *R*_on_ and *R*_off_ distributions corresponding to Figure 1e-h, respectively. The straight lines are the fitting lines according to the standard Weibull distributions. The values of Weibull slope (*β*) and scale factor (*R*_63%_) can be abstracted from the fitting. As shown in Table 1, the operation mode of CS-SET and VS-RESET presents the highest Weibull slopes of *R*_on_ and *R*_off_ distributions. In consequence, stable and uniform distributions of low and high resistance states can be obtained by the gradual SET and RESET operations.

**Table 1:** **The distributions of** *R*_on_ **and** *R*_off_ **under different operation modes**

**Operation**	**VS_SET and VS_RESET**		**CS_SET and CS_RESET**		**VS_SET and CS_RESET**		**CS_SET and VS_RESET**		**SP_P and SP_E**		**AP_P and AP_E**	
Parameters	*R*_on_	*R*_off_	*R*_on_	*R*_off_	*R*_on_	*R*_off_	*R*_on_	*R*_off_	*R*_on_	*R*_off_	*R*_on_	*R*_off_
*σ* (kΩ)	*0.716*	*3.7178*	*1.006*	*8.930*	*3.933*	*4.368*	*0.157*	*3.549*	*0.181*	*4.376*	*0.117*	*1.552*
*μ* (kΩ)	*1.218*	*25.338*	*1.927*	*29.154*	*3.513*	*24.494*	*0.673*	*32.051*	*0.575*	*10.708*	*0.453*	*12.687*
*σ*/*μ*	*0.588*	*0.147*	*0.522*	*0.306*	*1.119*	*0.178*	*0.234*	*0.111*	*0.315*	*0.409*	*0.268*	*0.122*
*β*	*5*	*9*	*3*	*3.4*	*2.1*	*6.7*	*6.5*	*10.5*	*3.5*	*5*	*5*	*10*
*R*_63%_(kΩ)	*1.274*	*26.425*	*1.945*	*32.233*	*2.582*	*26.049*	*0.700*	*33.010*	*0.637*	*10.427*	*0.491*	*13.976*

The comparison of four DC operation methods and two pulse operation methods on their controllability to the variation in Ti/HfO_2_/Pt RRAM device are listed in the above.

*σ* is the standard deviation, *μ* is the mean value, *σ*/*μ* is the coefficient of variation, and *β* and *R*_63%_ are the shape factor (or Weibull slop) and scale factor of the Weibull distributions of resistances, respectively.

**Figure 2:**
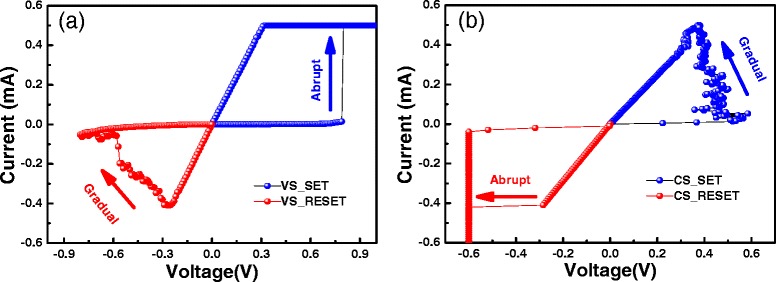
**The typical** ***I-V*** **curves of Cu/HfO**_**2**_ **/Pt RRAM device under two modes. (a)** Voltage sweep SET and RESET. **(b)** Current sweep SET and RESET.

We have also found that the current-controlled SET operation and voltage-controlled RESET operation are suitable for other kinds of RRAM devices. Figure 2a,b shows the typical *I-V* curves of electrochemical mechanism (ECM) device with Cu/HfO_2_/Pt structure measured by DC voltage sweep and DC current sweep, respectively. Similar effect in controlling the SET/RESET process can be achieved, i.e. gradual SET can be got by current sweep and RESET is progressive under voltage sweep.

**Figure 3:**
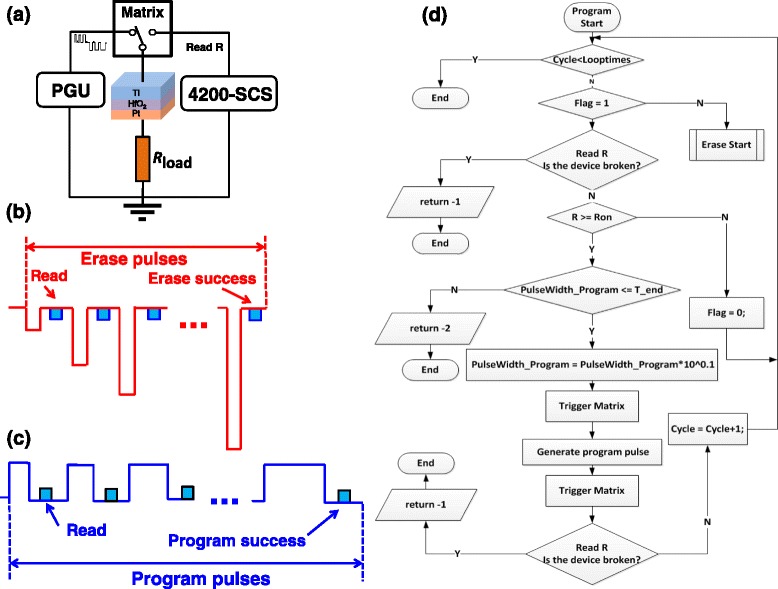
**The testing schematic of pulse operation method. (a)** The test circuit of our new pulse operation method. Pulses with width or height increased by the automatic procedure are applied to finish the program or erase operation, respectively. **(b)** Schematic diagram of one complete erase process with height-adjusting pulse operation. **(c)** Schematic diagram of one complete program cycle of width-adjusting pulse operation. **(d)** A detailed flow chart of the program method.

Considering the above SET/RESET characteristics in DC sweep, we use a new pulse operation to achieve the same effects. Figure 3a shows the test circuit of our new method. Pulses generated by PGU with width/height increased by an automatic procedure are applied on the RRAM device to finish the P/E operation. After each pulse, the connection is switched to 4200-SCS to carry out the DC read operation. Figure 3b,c shows in a more accurate way the schematic diagram of one complete erase process with height-adjusting pulse operation and one complete program process with width-adjusting pulse operation, respectively. Figure 3d provides the detailed flow chart of program test for our new pulse operation method. The width of the program pulses increases by around 1.1 times for each program-verify cycle. When the width exceeds the maximum pulse width *t*_end_, the procedure will be terminated. The flow chart of erase operation is similar to that of program test, and the height of erase pulse also increases about 1.1 times for each erase-verify cycle.

**Figure 4:**
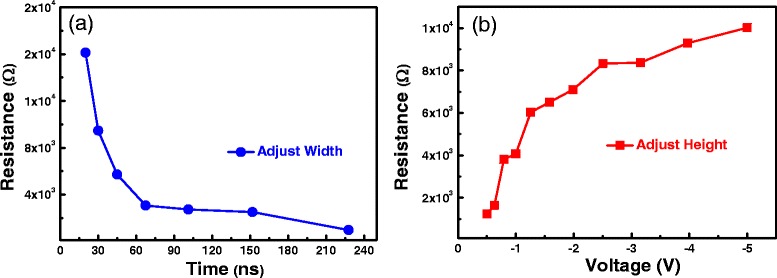
**The dependence of the resistance on the width/amplitude of P/E pulses.** The resistance gradually decreases with time by the width-adjusting program pulse operation **(a)** and gradually increases with voltage through the height-adjusting erase pulse operation **(b)**.

Figure 4 reveals the gradual change of the resistance using the width-adjusting program pulse operation and the height-adjusting erase pulse operation. The resistance gradually increases with time by the novel width-adjusting program pulse operation and gradually decreases with voltage through the height-adjusting erase pulse operation. Similar to the resistance adjusting by DC sweep method [22-25], our pulse operation methods can not only realize the gradual change of the resistances but can also be utilized to acquire multi-level storage of RRAM [26-29].

**Figure 5:**
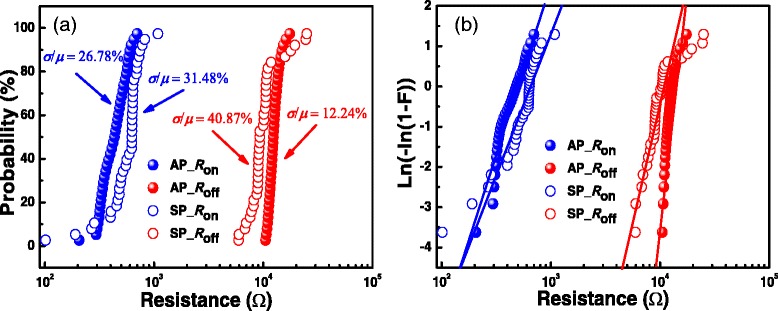
**The statistical distributions of** ***R*** **on and** ***R*** **off. (a)** The cumulative distributions of *R*_on_ and *R*_off_ obtained through width/height-adjusting pulses (AP) and single pulse (SP) operation. **(b)** The Weibull distributions of *R*_on_ and *R*_off_. *R*_on_ and *R*_off_ measured by our new pulse method have good uniformity.

During our new pulse operation, on-state and off-state resistance were respectively setup to the average value of *R*_on_ and *R*_off_ measured in DC sweep. After each pulse program or erase operation, the test circuit is switched by the matrix to 4200 to read the resistance (Figure 3a,d). When the resistance of the device is less than 30 Ω, we assume that the device is damaged, and the test will be stopped automatically. Figure 5 shows the comparison of the cumulative distributions of *R*_on_ and *R*_off_ measured by traditional single pulse operation and our new pulse operation. It can be seen that the coefficient of variation of *R*_on_ has changed from 31.48% to 26.78% and that of *R*_off_ has been greatly improved from 40.87% to 12.24%. Therefore, stable and uniform distributions of on-state and off-state resistance can be obtained by accurately controlling the SET/RESET switching by adjusting the pulse width/height, which is similar to the DC sweep mode.

**Figure 6:**
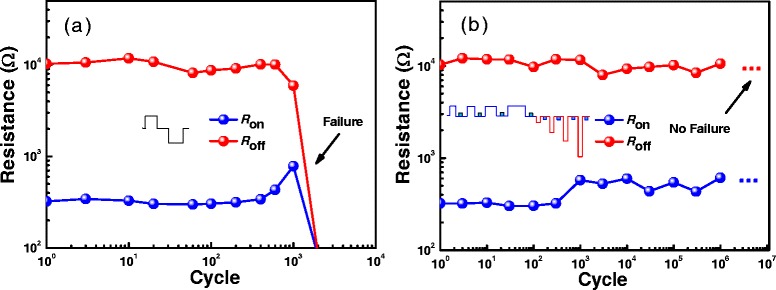
**Endurance characteristics of Ti/HfO**_**2**_ **/Pt RRAM device.** The comparison of endurance measured by traditional single pulse operation method **(a)** and width/height-adjusting pulse operation method **(b)** The endurance is significantly improved by the new pulse operation method.

Figure 6 shows the endurance characteristics measured by traditional single pulse operation and our new pulse operation. As can be seen from Figure 6a, the endurance tested with traditional pulse method is usually less than 10^3^ switching cycles, where the pulse amplitude/width is setup as 1 V/1 μs for program operation and 3 V/100 ns for erase operation. However, from Figure 6b, it is surprising that more than 10^6^ switching cycles have been obtained by our new pulse operation and the device still works well without failure. Here, the height and width of the program pulses are setup as 1 V and from 20 ns (initial) to 1 s (*t*_end_), respectively. The width and amplitude of the erase pulse are setup as 100 ns and from −0.5 V (initial) to −10 V (*V*_end_). The remarkable improvement in endurance is attributed to the appropriate program/erase operation in each cycle, without any inadequate operation and over-operation.

## Conclusions

We have investigated the impact of DC and pulse program/erase operation on the uniformity and endurance performances of Ti/HfO_2_/Pt-based RRAM device. Appropriate program/erase conditions are necessary to acquire the uniform resistive switching. A width-adjusting program and the height-adjusting erase pulse operation method are proposed. Our new method is advantageous to obtain the moderate program/erase operation in each cycle, without any inadequate operation and over operation. Thus, the endurance performance of the device is greatly improved. Based on our method, some technical solutions to improve the endurance of the RRAM can be developed.
